# A comparison of common programming languages used in bioinformatics

**DOI:** 10.1186/1471-2105-9-82

**Published:** 2008-02-05

**Authors:** Mathieu Fourment, Michael R Gillings

**Affiliations:** 1 Department of Biological Sciences, Macquarie University, Sydney, NSW 2109, Australia

## Abstract

**Background:**

The performance of different programming languages has previously been benchmarked using abstract mathematical algorithms, but not using standard bioinformatics algorithms. We compared the memory usage and speed of execution for three standard bioinformatics methods, implemented in programs using one of six different programming languages. Programs for the Sellers algorithm, the Neighbor-Joining tree construction algorithm and an algorithm for parsing BLAST file outputs were implemented in C, C++, C#, Java, Perl and Python.

**Results:**

Implementations in C and C++ were fastest and used the least memory. Programs in these languages generally contained more lines of code. Java and C# appeared to be a compromise between the flexibility of Perl and Python and the fast performance of C and C++. The relative performance of the tested languages did not change from Windows to Linux and no clear evidence of a faster operating system was found.

Source code and additional information are available from

**Conclusion:**

This benchmark provides a comparison of six commonly used programming languages under two different operating systems. The overall comparison shows that a developer should choose an appropriate language carefully, taking into account the performance expected and the library availability for each language.

## Background

Bioinformatic analyses involve a range of tasks and processes. Diverse programs have been written for various bioinformatics applications using every available language. Because of the size of bioinformatics datasets, computation time is not trivial, and efficiencies in computational speed are desirable. Comparisons of the algorithm accuracy of different programs that undertake similar tasks have been published [1-7] allowing assessment of the best algorithms to use for specific tasks. However, it is possible that the same program, written in different languages, or running under different operating systems, may exhibit significant differences in speed and efficiency. There is, at present, little direct data on the underlying speed and efficiency of equivalent algorithms written in different languages. While languages themselves have been benchmarked, such comparisons have not been done using algorithms that are relevant to bioinformatics [8].

A typical bioinformatics program reads FASTA files, holds the DNA sequences in memory, performs different computing tasks on the sequences, and finally writes the results to a file. Another common task in bioinformatics is text mining or text parsing. Large amounts of data can be generated in different formats. Because file formats can be different, linking programs in a pipeline is difficult, hence scripts are written to act as interfaces between programs performing the sequential parts of an analysis. Scripts are also used to extract information from large data files, thus enhancing the presentation of results. These quick scripts are usually implemented in Perl or Python. Consequently, any bioinformatics procedure has a number of areas where programming might be improved, these being: the space required to temporarily store data, the speed of computation, linkage between programs, and presentation of analyses.

In this paper we examined three commonly used tasks in biology, the Sellers algorithm [9] the Neighbor-Joining NJ algorithm [10] and a program parsing the output of BLAST [11]. In each case we tested the programs using different languages. This benchmark was conducted on both Linux and Windows, since the computer used had a dual boot. There were several reasons for this benchmarking exercise. We specifically wanted to determine if C would be faster than Java for performing recombination detection, which is an inherently difficult computational exercise. We also wanted to examine the memory requirements of each program/language combination, since although memory capacity increases constantly and hardware gets cheaper, the large datasets in bioinformatics analyses can be a problem for desktop computers. We also wanted to compare a script language, such as Perl, with the compiled languages Java and C. To complete the comparison, "rival" languages were also included. These included C++, C# and Python. The languages selected for this study were chosen on the basis that they are the most popular and frequently used for biological applications.

Python and Perl are often called script languages and when executed, are compiled in an intermediate representation without creating an intermediate file (syntax tree in Perl and byte code in Python) and then interpreted. Both languages use automatic memory management and have large free libraries. They are suitable for web scripting (e.g. CGI), parsing and pipeline implementation such as InterProScan [12].

C and C++ are fully compiled languages, suitable for system-intensive tasks.

Java and C# are semi-compiled languages using automatic memory management. A Java program is compiled in an intermediate-level code or bytecode then it is run by either an interpreter or compiler at runtime, in this case, the Java Virtual Machine (JVM). In C# the intermediate-level code is called Microsoft Intermediate Language and is run on the .NET Common Language Runtime engine.

Volunteer projects have produced libraries or modules for biologists. The most popular open source projects, which are incorporated in the Open Bioinformatics Foundation, are BioPerl, BioPython and BioJava [13].

## Results

The languages we investigated can be divided into 3 groups: The script group of Perl and Python; the semi-compiled group of Java and C#; and the compiled group of C and C++.

**Figure 1:**
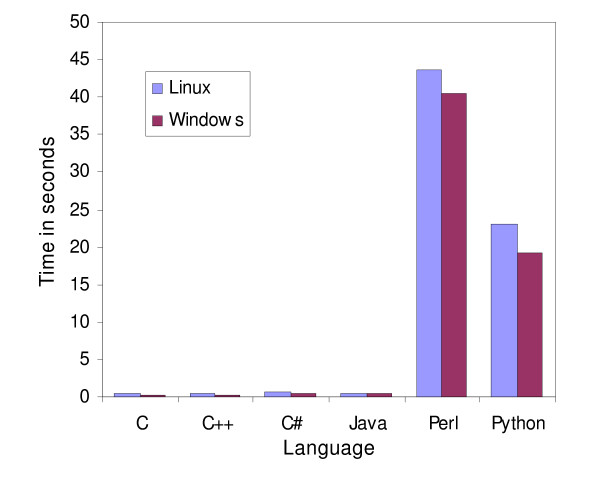
**Speed comparison of the global alignment program**. Speed comparison of the global alignment algorithm using a gap penalty of 10 implemented in C, C++, C#, Java, Perl and Python. The programs were run on Linux and Windows platforms. Two DNA sequences of 3216 bp and 3217 bp were used.

**Figure 2:**
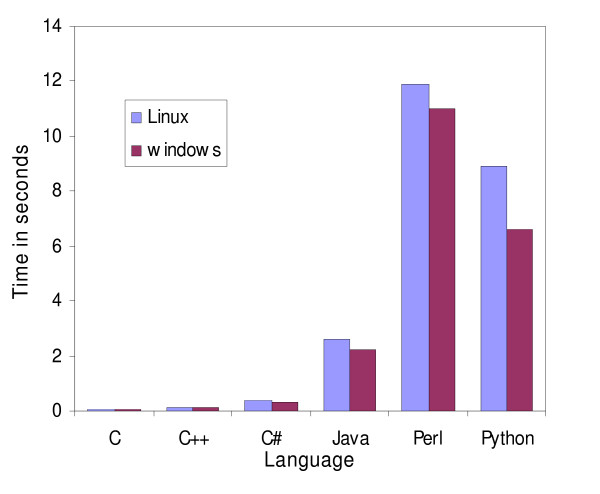
**Speed comparison of the Neighbor-Joining program**. Speed comparison of the Neighbor-Joining algorithm using the Jukes-Cantor evolutionary model implemented in C, C++, C#, Java, Perl and Python. The programs were run on Linux and Windows platforms. The input file was an alignment of 76 DNA sequences.

**Figure 3:**
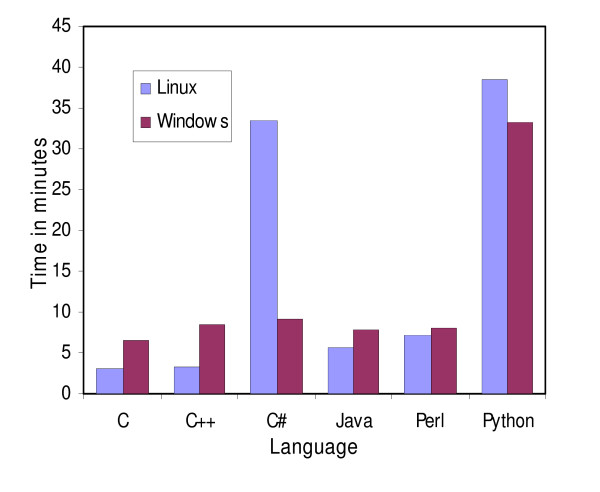
**Speed comparison of the BLAST parsing program**. Speed comparison of the BLAST parsing program implemented in C, C++, C#, Java, Perl and Python. The programs were run on Linux and Windows platforms. The input file was a 9.8 Gb file from a BLASTP run.

**Figure 4:**
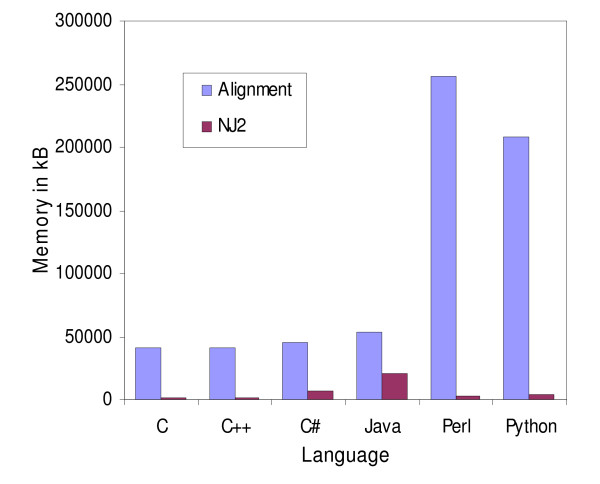
**Memory usage comparison of the Neighbor-Joining and global alignment programs**. Memory usage comparison for the Neighbor-Joining and global alignment programs implemented in C, C++, C#, Java, Perl and Python. The programs were run on a Linux platform.

Firstly we compared languages within groups, then we compared the groups to each other (Fig. 1, 2, 3, 4), and finally we compared speed performance between Windows and Linux. In this paper we will refer to ease of coding as the number of coding lines needed to write a program, taking into account the availability of libraries, which is a factor in the number of coding lines needed for compiling a program.

### Perl versus Python

Perl clearly outperformed Python for I/O operations. Perl was three times as fast as Python when reading a FASTA file and needed half of the space to store the sequences in memory (Fig 4). From the results of the global alignment and NJ programs Python appeared to have better character string manipulation capabilities than Perl. Even though the NJ program required reading a file, where Python did not perform well compared to Perl (Fig 2), the computation of the dissimilarity matrix was actually the most discriminating task, since more than 90% of processing time was taken up by this step for every language except C, where it took up 75% of processing time.

Python was the worst performer for parsing a BLAST file (Fig 3), taking more than 38 minutes to process the file compared to Perl, which took only 7.28 minutes. This difference did not arise from any inability of Python to handle large files, since it took only 3.2 minutes to read the file without processing the lines. Perl accomplished the same task in only 1.4 minutes.

Perl emphasizes support for common application-oriented tasks, by having built-in regular expressions, file scanning and report generating features. Python emphasizes support for common programming methodologies such as data structure design and object-oriented programming.

### Java versus C#

C# appeared to require less memory than Java for holding strings in memory, as demonstrated when reading DNA sequences from a file (Fig 4). C# also needed less time to read this type of file. Interestingly, Java was slightly faster in the global alignment program (Fig 1) but much slower in the NJ program (Fig 2). Java regular expression implementation appeared to outperform C# (Fig 3). This difference did not arise through any inability of C# to handle large files, since it read these files faster than Java did. Java needed 3.2 minutes whereas C# took only 2.8 minutes to read the same file.

### C versus C++

The performance of C and C++ was very similar (Fig 1, 2, 3, 4). This is perhaps not surprising since C++ is an extension of C. When a C program was compiled with the C++ compiler we obtained near-identical results, but when C++ standard libraries (ie. character strings) were used, the performances tended to slightly deteriorate. It is important to note that tokenization was twice as fast as regular expressions for parsing the same BLAST file, but it took more time to write the program using tokens.

### Group versus group

The global alignment example demonstrated that the semi-compiled languages (Java and C#) were nearly as fast as the compiled group (C and C++), whereas the interpreted languages (Perl and Python) were sixty-fold slower (Fig 1). In the NJ program, the performance of C# was similar to C and C++, while Java took significantly more time (Fig 2).

The biggest drawback for semi-compiled languages is their memory usage, since they required about 20 times more memory than C and 3 times more memory than Perl (Fig 4).

Java and C# appeared to be a compromise between the speed of C/C++ and the ease of coding of Perl/Python.

Surprisingly, Java performed better than Perl during the regular expression benchmark.

In Java it is possible to embed C code to enhance the efficiency of a program using Java Native Interface (JNI) extensions. The equivalent in Perl would be the eXternal Subroutine (XS) extension. For example, the core of the NJ program was written in Perl, but when the subroutine calculating a pairwise comparison was written in C, it sped up the program from 11.8 seconds to 0.29 seconds. JNI improved this speed to a lesser extent, from 2.58 seconds to 0.71 seconds. Any loss of portability was compensated for by the gain in performance, since there was no need to rewrite the entire program.

### Windows versus Linux

The relative performance of the tested languages did not change on Windows but the overall performance changed depending on the program compared. Only C# and Python appeared consistently faster in every program on Windows. In the global alignment program all the implementations performed better in the Windows environment (Fig 1). In the NJ (Fig 2) and the BLAST parser (Fig 3), C and C++ were both slower on Windows whereas on Windows, Java and Perl were faster in the NJ example (Fig 2) but slower in the BLAST parser example.

The comparison of Linux and Windows has to be carefully interpreted, since the compiler implementations are different, as well as the operating system running them. In the end, speed and memory usage are the critical factors, since the user is looking for performance in the programs, not more generally in the OS or compilers.

### Case study: BLAST server

A fast and memory efficient program can make a significant difference when running on a public server such as BLAST, which is queried millions of time a day. The obvious choice for such a computer intensive program was to use C with Perl CGI for the web interface. If we consider that Perl was nearly 60 times slower than C in the global alignment benchmark and that a query sequence of 3500 nucleotides against the non-redundant database took roughly 10 seconds (including the transfer over the web), then if the query is submitted a million times during the day, the total computation time would have increased 60 fold, taking considerably more server time. The same observation would apply for the memory usage. After choosing the appropriate programming language, it is also important to keep improving the base algorithm. New algorithms for analyzing phylogenetic relationships have reduced computing time from weeks to days, or even hours [14].

## Discussion

All the programs examined here were written by the same programmer with different levels of experience in Java, Perl and C++. The other languages were implemented while learning them. Even though the semantics of these languages is similar, since C influenced C++, C#, Java, Perl and Python directly or indirectly, the philosophy of some of the languages is different and programs should be implemented according to the language paradigm. For example, Perl programmers favor hash tables to arrays, coupled with a loop which is more widely used in C. It is also important to keep in mind that the hash function can be costly when adding a new value and the memory allocated would be larger than an array containing the same number of elements. The advantage of a hash table is the speed in retrieving some data, but when the programmer needs to examine sequentially all the values in the hash table, then a hash table should be avoided because of the extra cost occurring when adding the key-value pair. In the Perl NJ algorithm an implementation using an array to store the sequences appeared to be faster and more memory efficient than a program using a hash table. Hence no hashtable was used in this benchmark. There is an important tradeoff between performance and convenience. Perl and Python allow reading and loading a file in memory in one statement. While this approach is convenient compared to reading and processing a file line by line, the operating system could start swapping memory out, thus slowing everything down.

Object creation, garbage collection and memory recycling are costly in terms of CPU and memory usage, hence some precautions should be taken when creating objects and the number of objects should be reduced as much as possible. To prevent memory leaks or heavy applications, objects should also be reused when possible and immutable objects such as the String object in Java should be avoided especially when temporary objects are created in frequently used routines. C# and Java have a higher memory-size penalty for objects than other object oriented languages such as C++ due to their ability to use reflection. Reflection is a powerful tool that contributes to the flexibility of these two languages. However this feature should only be used when needed, since reflection method calls have a substantial performance overhead, make the code harder to understand and errors are found at runtime instead of compile-time.

The way objects are accessed and stored in memory influences the performance of each language. C++, C# and Java store objects as a block of data and access them via constant offsets, whereas objects in Python are implemented as hash tables. There are several ways to create objects in Perl. Different data structures can be used, but most programmers use hashes, even though arrays are faster, prevent attribute collisions and take less memory.

It is worth noting that the Perl implementation of the NJ algorithm was substantially improved by converting each sequence to an array instead of using the substr function on the string of characters for computing the similarity matrix. Although the program was 10% faster, the memory footprint showed a ten-fold increase.

### Expressiveness

**Figure 5:**
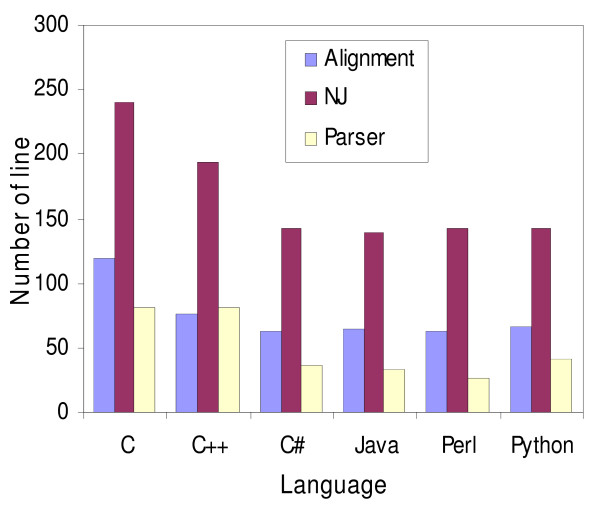
**Number of lines for each program**. Number of lines for the global alignment, BLAST parser and Neighbor-Joining programs implemented in C, C++, C#, Java, Perl and Python.

The number of lines in a program varies from one programmer to another, and also on their willingness to shorten the code to the detriment of readability. It is important to emphasize that it is hardly possible to find a correlation between expressiveness and performance. Nevertheless, a noticeable difference was observed (Fig. 5), especially with regular expressions. In Perl, a unique statement can be used to detect a pattern and the captured pattern is retrieved with the special variable $1, whereas in Java the programmer has to instantiate a Pattern object which is a compiled representation of the regular expression, then create a Matcher object which performs match operations on a character sequence by interpreting the pattern object. The following examples illustrate the retrieval of a GI number from a FASTA file:

Perl:

print $1 if($string =~/^>gi\ |(\ d{3,})/);

Java:

Pattern p = Pattern.compile("^>gi\ |(\ d{3,})");

Matcher m = p.matcher(string);

if(m.find()) System.out.print(m.group(1));

Language philosophies often explain differences in the relative expressiveness and readability of languages. For example, the philosophy of Python is to take the clearest, simplest and most straightforward approach to writing a program, and to accept the resulting performance penalty. Whereas Perl gives more freedom to the programmer resulting, in some cases, in programs that are unreadable for non Perl programmers.

Factors such as performance and memory usage are important, but need not be the sole determinant when choosing a language. Since time management is also an important factor, a language can be chosen for its library, future scalability, active community and interface to other languages.

While it is hard to define a learning curve for each language, advantages and disadvantages of each language can be found. Memory management such as memory allocation makes tasks easier for Java, C#, Perl, Python and Perl programmers, even though memory usage should always be under scrutiny. The use of pointers in C and C++ can be overwhelming for a learner, and it may take some time to master their use. Python is designed to be a highly readable language, frequently using English keywords, whereas the five other languages use punctuation.

Platform independence can be a factor for choosing a language and can also facilitate its learning. Java uses a virtual machine to run on different operating systems and since Perl and Python are stored in a text file and not in a binary file these scripts can be run on any computer having the appropriate interpreter.

The standard library diversity and size of Java, Python and C# are a major advantage compared to the other languages,. including sets of classes to create graphical interfaces, data structures (vectors, hash tables, stacks, queues), regular expression, database access and networking.

Although the Perl standard library appears smaller compared to Java, Python and C#'s, it benefits from a large community of programmers creating modules gathered by the CPAN network [15].

C has a very limited standard library supporting input and output streams, memory allocation, mathematics, character strings, and time values. The C++ library contains the C library as well as a class to manipulate string of characters and the Standard Template Library containing containers or data structures such as algorithms, an improved String library and input/output stream libraries.

As shown previously in the global alignment example, Java and Perl can communicate with a program written in C, speeding up the program using JNI and XS respectively. For example, if a computer intensive command based program written in C needs a graphical interface, an easy solution would be to use the Swing library and the JNI framework instead of rewriting the whole program in Java. The possibility of a language to language interface is useful when some code is already written in one of them.

As well as C, the JNI framework allows Java to interact with C++ and Fortran. The downside of this approach is the loss of portability. Java can also interact with Python using Jython, which is an implementation of Python in Java.

Some bioinformatics tasks, such as loading a FASTA file or parsing a BLAST file, are so frequently used that it makes sense to reuse bits of code by creating libraries or modules. Many programmers produce modules for the bioinformatics community and make them available through their websites. The most widely used libraries are BioPerl, BioPython and BioJava. These Open Source projects belong to the Open Bioinformatics Foundation and they provide toolkits with multiple functionality that make it easier to create customized pipelines or analysis.

Unfortunately libraries with this level of organization and diversity are not available for C, C++ and C# even though some small libraries are available independently [16,17]

In this paper we focused on the performance of languages, not on the performance of the algorithm implementations. For example the speed of the alignment program could be improved by using a one-dimension array of size n × m instead of a 2-dimensional matrix of size n × m. This approach would speed up the memory allocation, but this was not the goal of this benchmark.

In this paper, to find the fastest implementations we used profiling tools included in libraries or using compilation/execution options. Profiling provides important information about applications, such as memory usage and the fraction of time spent in each function. By using profiling and a trial-and-error approach programs were significantly improved. We used the Devel::DProf library in Perl, the Xprof option in Java, the cProfile module in Python, the default profiler in C# using -profile = default and the -pg compiler option coupled with the gprof program in C and C++.

## Conclusion

As expected, C was the best performer in this benchmark, in terms of both speed and memory usage. But to achieve such performances generally requires more code because of the reduced standard library. This benchmark is only a preliminary test involving a limited number of analysis types. Comparisons using different programs may change the relative performance of the languages. Graphic interfaces are very important in biology, hence it would be interesting to compare the libraries available.

The best choice of language for a task would be according to the original philosophy, keeping in mind that Java is portable web oriented language, Perl is a powerful script language, Python is an easily coded language and C and C++ are efficient languages used in operating systems and drivers.

Performances can also vary depending on the compiler and version used. Sun is constantly improving the Java compiler and interpreter and other JVM implementations are also available such as Kaffe [18] and IBM's.

The primary motivation for this benchmark was to compare a recombination detection program in Java and C. The recombination program written in Java ran in 11 minutes and the new version in C ran in 9 minutes with the same dataset and the same parameters. This test involved some hundreds of sequences and a single gene target. Given the rapid improvements in high throughput sequencing technologies, it is likely that tests involving orders of magnitude more sequences will be conducted in the future. Savings in computing time will be essential for such analyses to be efficient.

## Methods

### Benchmark design

During the benchmarking process, unnecessary services were disabled. Each program was run three times and the minimum time and memory usage were recorded.

C and C++ programs were optimized with the flag -O3. No multi-threading was used explicitly to write the programs. Compilers or interpreters are described in Table 1.

**Table 1:** Language list with respective compiler or interpreter name and version

	Linux		Windows	
Language	Compiler/interpreter	version	Compiler/interpreter	version

C	GNU gcc	4.1.1	gcc	3.4.2
C++	GNU g++	4.1.1	g++	3.4.2
C#	gmcs/mono	1.1.17.1	.NET csc	2.0.50727
Java	Sun JDK javac/java	1.5.0_09	Sun JDK javac/java	1.5.0_12
Perl	Perl	5.8.8	Active state perl	5.8.8
Python	Python	2.4.4	python	2.5.1

The speed of processing was timed using the GNU program 'time' which is present in most of the Linux distributions. In this paper we present only the user time from the output. Profiling was also used to inspect the time repartition and memory allocation. On Windows, a simple Perl script launching the programs with the option -Dprof was used to time all processes. The memory usage was measured on Linux using the command "grep Vm/proc/pid/status", only for the NJ and global alignment programs since parsing does not use much memory.

All the programs were run on the following machine with a dual boot Linux/Windows:

Linux: Fedora core 7, kernel 2.6.21-1.3228

Windows: Windows XP professional, Version 2002, service pack 2

Intel(R) Core(TM)2 CPU 6400 @ 2.13 GHz

4 GB DDR2 memory

250 GB hard drive

## Algorithms

### Sellers algorithm [9]

The Sellers algorithm is a simple global sequence alignment method using a dynamic programming approach with a gap penalty. Scores for aligned characters are computed (see equation 1) and stored in a similarity matrix F with a linear gap penalty d, and where s(i, j) is the substitution score for character i and j.

(1) $$F(i,j)=\max\{\begin{array}{l} F(i-1,j)+d\\ F(i-1,j-1)+s(i,j)\\ F(i,j-1)+d \end{array}$$

In this example a two-dimensional matrix is used to store the highest scores with the top left corner representing the beginning of the sequences and the bottom right corner representing the end of the sequences, which is the starting point of the alignment. For sequences of sizes n and m, the running time of the algorithm is O(nm) and the amount of memory used is in O(nm). This program requires reading sequences from FASTA files, initializing a two-dimensional matrix, character comparison and character string concatenation. A sub-sequence of 3216 nucleotides from the L segment of a *Hantavirus *genome [GeneBank:AJ005637] was used and then deletions, insertions and SNPs were introduced manually along the sequence at random to generate a second sequence of 3217 nucleotides. The sequences were not read from a file but were hard coded to avoid input streams, and thus focus entirely on memory allocation and character string manipulations.

### Neighbor-Joining method [10]

NJ is a distance-based algorithm for constructing phylogenetic trees and is probably the most widely used distance based method. The method uses the minimum evolutionary criterion and starts by assuming a bush-like tree that has no internal branches. Then it combines node i and node j that minimizes equation 2 where r is the current number of nodes and d(i, j) is the distance between i and j. At each stage in the process two terminal nodes are replaced by one new node. This process is repeated until two nodes are separated by one branch.

(2) $$Q(i,j)=(r-2)d(i,j)-\sum_{k=1}^{r}d(i,k)-\sum_{k=1}^{r}d(j,k)$$

The running time of the algorithm is O(n^3^) and the amount of memory used is in O(n^2^).

This program requires reading sequences from FASTA files, initializing a two-dimensional dissimilarity matrix from a pairwise comparison of the DNA sequences, and finally performing the clustering algorithm. Memory allocation, input and output streams, character string manipulation and tree building are the main components of this program. The nodes of the tree were implemented as structures in C and C++, as objects in C#, Java and Python and anonymous hash tables in Perl.

The most computer intensive component of this program is the pairwise comparison used to compute the dissimilarity matrix. In the test example 76 *Hantavirus *segment L sequences were used with an overall alignment length of 6580 nucleotides.

### Basic Local Alignment Search Tool parsing BLAST [11]

BLAST is a tool calculating sequence similarities between a query sequence and sequences lodged in a specially formatted database. It uses a lookup table to match words and a local alignment method to extend them. In the output one can find the query aligned to a similar sequence and statistics about the significance of the alignment.

BLAST results can be as large as several gigabytes and a program is usually needed to parse the interesting parts or to feed another program. For example, numerous Gene Ontology [19] programs use BLAST outputs to assign GO terms to unknown sequences.

Tokenization can be used to parse BLAST result files but this can be tedious and requires a good knowledge of the structure of the input. Regular expressions make this kind of task easier, with the only inconvenience being potential loss in speed. The program parses and prints in a one line comma separated file, the name of the sequence, containing the gene identifier, bit score, identity percentage, e-value and positive matches. In the example we tested, the output was redirect to/dev/null on Linux and NUL on Windows.

The test program made use of input streams and regular expressions. A program using tokenization was written in C as a control to benchmark regular expressions.

A 9.8 Gb file from a BLASTP search with a *Caenorhabditis elegans *sequence (accession number [GeneBank:ABD75716] was used in this program. Our program did not aim to compare the regular expression performances of each language but the overall speed of such a task. Since the regular expressions used are quite simple and are applied to relatively short strings of characters (a line in a BLAST file is usually not longer than 80 characters) the program will spend more time reading the file than actually parsing it.

To read such a large file and overcome the 2 GB file size limitation the flags "-D LARGEFILE_SOURCE -D_FILE_OFFSET_BITS = 64" were used when compiling the C and C++ source code.

The Perl Compatible Regular Expression [20] library was used for C and C++ since their standard library does not implement built-in regular expressions.

## Abbreviations

BLAST, Basic Local Alignment Search Tool; CGI, Common Gateway Interface; CPAN, Comprehensive Perl Archive Network; CPU, Central Processing Unit; DNA, Deoxyribo Nucleic Acid; FASTA, FAST-All; GI, Geninfo Identifier; GO, Gene Ontology; I/O, input and output; JNI, Java Native Interface; JVM, Java Virtual Machine; NJ, Neighbor Joining; SNP, Single Nucleotide Polymorphism; XS, eXternal Subroutine.

## Authors' contributions

MF participated in the design of the study and in the implementation of the programs. MRG participated in the design and coordination of the study and helped to draft the manuscript. All authors read and approved the final manuscript.
